# Rift Valley Fever at Europe’s Risk Frontier: Climate, Vectors, and One Health Preparedness

**DOI:** 10.3390/vetsci13090873

**Published:** 2026-08-27

**Authors:** Koycho Koev

**Affiliations:** Faculty of Veterinary Medicine, Department of Veterinary Microbiology, Infectious and Parasitic Diseases, Trakia University, 6000 Stara Zagora, Bulgaria; koycho.koev@trakia-uni.bg

**Keywords:** Rift Valley fever, Rift Valley fever virus, RVFV, zoonosis, vector-borne disease, Europe, Mediterranean region, climate change, One Health, surveillance, preparedness

## Abstract

**Background:** Rift Valley fever (RVF) is a livestock-amplified, mosquito-borne zoonosis caused by Rift Valley fever virus (RVFV). Continental Europe has no established RVFV transmission, but a rare introduction could have major consequences if susceptible ruminants amplify infection before detection. **Methodology:** This critical narrative review synthesizes 69 scientific and institutional sources identified through structured PubMed/MEDLINE and Europe PMC searches, citation verification, and qualitative appraisal. **Findings:** European preparedness should distinguish probability from impact and weight evidence by decision value. Direct virological or acute animal evidence has the highest escalation value; serology, vector competence, environmental suitability, and pathway models mainly indicate where investigation should intensify. The main vulnerability is delayed recognition when animal movement or transported vector exposure coincides with seasonal receptivity and uneven surveillance capacity. **Conclusions:** The review develops a conditional convergence framework linking evidence strength to baseline vigilance, targeted investigation, and emergency response. Preparedness should prioritize differential diagnosis of livestock abortions and neonatal deaths, pathway-specific risk reduction, trigger-based sampling, occupational protection, and predefined vaccination and movement control decisions.

## Simple Summary

Rift Valley fever affects cattle, sheep, goats, camels and people. People are usually infected while handling sick animals, aborted material, carcasses, blood or fresh animal products; mosquitoes mainly spread the virus among animals. Continental Europe has no established disease, but a rare introduction could have serious consequences if it reached unexposed herds during a mosquito-active season and was recognized late. The central question is how Europe should prepare before such an event. Routine preparedness should keep veterinarians alert, maintain laboratory access, and ensure reporting of abortions or newborn deaths. Investigation should intensify when unusual animal disease occurs in a place and season where animal movement, mosquitoes and worker exposure make infection plausible. Emergency action should then focus on animal testing, movement control, worker protection and vaccination planning. This staged approach can protect animals and people while avoiding broad measures when evidence is weak.

## 1. Introduction

Rift Valley fever (RVF) sits at the intersection of veterinary infectious disease and public health because mosquito amplification, high livestock viraemia and occupational exposure are biologically linked. Its etiological agent, Rift Valley fever virus (RVFV), is a tri-segmented RNA phlebovirus [1]. Veterinary and clinical reviews describe a disease system in which ruminant viraemia produces fetal loss, neonatal disease and hepatic injury, while humans are exposed mainly during contact with infected animals or fresh tissues [2,3]. In food-animal medicine, severe neonatal disease and abortion clusters in pregnant ruminants are the dominant outbreak signals [4]. Human disease is usually febrile but can progress to ocular, neurological, hepatic or haemorrhagic complications [2,3]. These links matter for surveillance because animal amplification can precede many occupational exposures, yet weak reporting or laboratory access may allow human cases to be recognized before animal outbreaks are formally documented.

Pregnancy-related RVF evidence must be separated by host species and by epidemiological scale. In livestock, abortion storms, stillborn fetuses, neonatal losses, and exposure to placental membranes or other tissues expelled during abortion are herd-level veterinary events and major occupational exposure hazards [4]. RVFV can target the maternal–fetal interface in ovine and human placental tissues [5]. In humans, RVF-related miscarriage has been confirmed, although the available evidence supports a serious clinical association distinct from the population-level pattern of ruminant abortion storms. A Sudanese cross-sectional hospital study found miscarriage in 15/28 (54%) febrile pregnant women with acute RVFV infection compared with 12/102 (12%) RVFV-negative women, with acute RVFV infection independently associated with miscarriage (odds ratio 7.4, 95% confidence interval 2.7–20.1) [6]. This study supports a clinically important human pregnancy signal, while its small number of acute RVFV-positive pregnant women and hospital-based febrile sampling frame limit population-level comparison with ruminant herd outbreaks. Therefore, livestock reproductive losses are treated here as veterinary sentinel and exposure events, whereas human miscarriage is treated as a serious but more sparsely documented clinical outcome.

Recognized human RVF is usually acquired through direct contact with infected blood, fetal or placental material, carcasses and slaughter-related fluids in people who work closely with livestock [2,3]. Mosquito-borne human infection can occur, but reported outbreaks often center on occupational exposure [7]. This distinction matters because mosquitoes maintain livestock–vector amplification, whereas many human cases arise during animal care, slaughter or disposal of abortion material. Prevention should follow that exposure sequence. Animal health surveillance and laboratory confirmation identify the amplifying event [4]. Occupational protection and farm biosecurity reduce direct exposure [7]. Vector, climate, and movement pathway evidence then defines where investigation should intensify.

For Europe, the central scientific problem is the difference between low estimated incursion probability and high potential consequence after delayed recognition in naive livestock. Early European assessments framed RVF as a plausible future threat on a continent without established disease [8]. Later public health analysis made the same issue relevant for the European Union (EU) [9], and comparative assessment for the EU and United States separated entry probability from establishment conditions [10]. The European Food Safety Authority (EFSA) currently assesses EU introduction risk as very low under existing controls [11]. Europe’s risk frontier denotes the preparedness zone in which entry pathways, seasonal receptivity, susceptible livestock and surveillance capacity begin to overlap. A very-low-risk estimate is therefore a probability statement, not a statement that impact would be low. Preparedness becomes justified when evidence suggests that introduction, receptivity and delayed detection could converge.

Climate and environmental evidence is most useful when it changes where and when surveillance is intensified. Temperature, rainfall and land use change alter livestock disease hazards [12], but RVF examples show that environmental suitability becomes operational only when it intersects with vector ecology and animal movement. Madagascar linked amplification to rainfall, local vectors and livestock movement [13]; suitability mapping identified seasonal surveillance windows [14]; and the 2000 Southwestern Arabian outbreak showed that wet conditions required prior viral introduction before livestock amplification became visible [15]. Global health syntheses and interepidemic modeling extend this point by connecting prevention to hydrology, demography, and vector ecology changing at different rates [16,17,18]. For Europe, this evidence supports trigger-based timing and localization of animal health and entomological investigation.

Accordingly, the central objective is to determine which forms of RVFV evidence should change preparedness decisions in Europe. Virological and pathological evidence defines what must be detected. NSs-mediated suppression of host transcription and interferon production explains virulence [19,20], and neonatal-lamb pathology explains why hepatic injury, abortion and neonatal mortality are central animal health endpoints [21,22]. Unresolved questions about RVFV maintenance and transmission justify evidence triage [23]. Conditional convergence defines a surveillance decision framework in which escalation is based on the spatial and temporal alignment of independent but complementary signals, rather than on any single finding. In this framework, evidence of plausible viral entry or compatible animal disease becomes operationally actionable when it coincides with receptive vector–environment conditions, susceptible livestock populations, limited early-detection capacity, or increased occupational exposure risk, and may support targeted veterinary sampling, movement tracing, focused vector investigation, worker protection measures, and laboratory confirmation. The synthesis therefore moves from endpoint appraisal to entry-route interpretation, then to scenario-based preparedness and resource allocation.

## 2. Materials and Methods

### 2.1. Review Design and Objective

This critical narrative review was designed to support structured literature searching, citation verification, and qualitative evidence synthesis. It was not designed or reported as a systematic review, scoping review, meta-analysis, or formal evidence map.

The review question was how Rift Valley fever virus (RVFV) etiology, pathogenesis, animal–human transmission, vector competence, climate-sensitive environmental suitability, introduction pathways, and operational preparedness evidence should be interpreted when evaluating RVF as a low-probability but potentially high-impact infectious disease and preventive medicine concern for Europe.

A narrative design was selected because the review integrates heterogeneous evidence from virology, veterinary pathology, human clinical disease, outbreak investigation, seroepidemiology, experimental vector competence, ecological and transmission modeling, risk assessment, diagnostics, vaccination, and One Health preparedness policy.

### 2.2. Information Sources and Search Date

Structured searches were conducted in PubMed/MEDLINE and Europe PMC. Bibliographic identity and citation authenticity were checked through PubMed records, Europe PMC records, digital object identifier (DOI)/Crossref-linked metadata, publisher pages, and official institutional records. Targeted official sources from the European Food Safety Authority (EFSA), the European Centre for Disease Prevention and Control (ECDC), the World Health Organization (WHO), the World Organisation for Animal Health (WOAH), and the Food and Agriculture Organization of the United Nations (FAO) were used when they directly supported disease status, European risk assessment, surveillance, diagnostics, vaccination, control measures, or preparedness interpretation.

Searches covered records available from database inception to 9 August 2026, with no lower publication date limit. No publication date restriction or language filter was applied at the search stage because historical veterinary pathology, vaccine safety, and vector competence studies remain relevant to RVFV pathogenesis and preparedness interpretation. Sources were retained only when the available English abstract, full text, publisher record, or official institutional record allowed reliable interpretation and citation verification. Recent literature was prioritized when addressing rapidly changing topics, including European risk assessment, environmental and transmission modeling, vector competence, diagnostics, vaccines, and surveillance preparedness.

### 2.3. Database-Specific Search Strategy

The PubMed/MEDLINE query used the disease block (“Rift Valley Fever”[MeSH Terms] OR “Rift Valley fever”[Title/Abstract] OR “Rift Valley fever virus”[Title/Abstract] OR RVF[Title/Abstract] OR RVFV[Title/Abstract]).

This disease block was combined with the title/abstract concept block (Europe[Title/Abstract] OR European[Title/Abstract] OR “European Union”[Title/Abstract] OR Mediterranean[Title/Abstract] OR “North Africa”[Title/Abstract] OR “Middle East”[Title/Abstract] OR “risk assessment”[Title/Abstract] OR preparedness[Title/Abstract] OR surveillance[Title/Abstract] OR “One Health”[Title/Abstract] OR introduction[Title/Abstract] OR incursion[Title/Abstract] OR establishment[Title/Abstract] OR import*[Title/Abstract] OR trade[Title/Abstract] OR “animal movement”[Title/Abstract] OR “transported mosquitoes”[Title/Abstract] OR mosquito*[Title/Abstract] OR vector*[Title/Abstract] OR “vector competence”[Title/Abstract] OR *Aedes*[Title/Abstract] OR *Culex*[Title/Abstract] OR transovarial[Title/Abstract] OR “vertical transmission”[Title/Abstract] OR climate[Title/Abstract] OR temperature[Title/Abstract] OR pathogenesis[Title/Abstract] OR hepatic[Title/Abstract] OR abortion[Title/Abstract] OR fetal[Title/Abstract] OR neonatal[Title/Abstract] OR haemorrhagic[Title/Abstract] OR diagnostic*[Title/Abstract] OR serolog*[Title/Abstract] OR PCR[Title/Abstract] OR “point-of-care”[Title/Abstract] OR vaccine*[Title/Abstract] OR vaccination[Title/Abstract] OR DIVA[Title/Abstract] OR biosecurity[Title/Abstract] OR control[Title/Abstract]).

The Europe PMC query used the disease block (TITLE_ABS:”Rift Valley fever” OR TITLE_ABS:”Rift Valley fever virus” OR TITLE_ABS:RVF OR TITLE_ABS:RVFV).

This disease block was combined with the concept block (TITLE_ABS:Europe OR TITLE_ABS:European OR TITLE_ABS:”European Union” OR TITLE_ABS:Mediterranean OR TITLE_ABS:”North Africa” OR TITLE_ABS:”Middle East” OR TITLE_ABS:”risk assessment” OR TITLE_ABS:preparedness OR TITLE_ABS:surveillance OR TITLE_ABS:”One Health” OR TITLE_ABS:introduction OR TITLE_ABS:incursion OR TITLE_ABS:establishment OR TITLE_ABS:import OR TITLE_ABS:trade OR TITLE_ABS:”animal movement” OR TITLE_ABS:”transported mosquitoes” OR TITLE_ABS:mosquito OR TITLE_ABS:mosquitoes OR TITLE_ABS:vector OR TITLE_ABS:vectors OR TITLE_ABS:”vector competence” OR TITLE_ABS:*Aedes* OR TITLE_ABS:*Culex* OR TITLE_ABS:transovarial OR TITLE_ABS:”vertical transmission” OR TITLE_ABS:climate OR TITLE_ABS:temperature OR TITLE_ABS:pathogenesis OR TITLE_ABS:hepatic OR TITLE_ABS:abortion OR TITLE_ABS:fetal OR TITLE_ABS:neonatal OR TITLE_ABS:haemorrhagic OR TITLE_ABS:diagnostic OR TITLE_ABS:diagnostics OR TITLE_ABS:serology OR TITLE_ABS:serological OR TITLE_ABS:PCR OR TITLE_ABS:”point-of-care” OR TITLE_ABS:vaccine OR TITLE_ABS:vaccines OR TITLE_ABS:vaccination OR TITLE_ABS:DIVA OR TITLE_ABS:biosecurity OR TITLE_ABS:control).

The searches were intentionally broad at the retrieval stage. Screening, eligibility assessment, and qualitative evidence appraisal were then used to determine relevance to the European RVF preparedness question and the evidentiary weight assigned to each retained source.

### 2.4. Record Management, Eligibility Criteria, and Source Selection

To document retrieval volume, the PubMed/MEDLINE strategy was run as five broad grouped searches reflecting the major evidence domains of the review: Europe, preparedness, and vector–climate context (1678 records); pathogenesis and disease outcomes (906 records); vectors and vector–virus interaction (1232 records); European and connected pathway or modeling context (1233 records); and surveillance, diagnostics, vaccination, control, and biosecurity (1487 records).

After deduplication across PubMed/MEDLINE grouped searches, 2317 unique PubMed records remained. Europe PMC was searched with the same grouped domains and yielded 1467, 956, 1230, 855, and 1578 records, respectively; 2386 unique Europe PMC records remained after within-database deduplication. Cross-database deduplication was performed using PubMed identifier (PMID), DOI, or source-specific record identifier when PMID or DOI was unavailable. A total of 2150 Europe PMC records duplicated PubMed/MEDLINE records, and 236 Europe PMC-only records were retained in the screening pool, leaving 2553 unique bibliographic records for title/abstract relevance screening.

Records were eligible when they addressed RVFV or RVF in relation to viral etiology, genome organization, virulence mechanisms, hepatic, placental, fetal or neonatal pathology, animal disease, human disease, occupational exposure, animal–human transmission, vector competence, vector–virus interactions, transovarial or vertical transmission, interepidemic maintenance, climate or environmental drivers, introduction pathways, European or Mediterranean receptivity, surveillance, diagnostics, vaccination, biosecurity, or One Health preparedness.

Eligible source types included peer-reviewed primary studies, outbreak investigations, seroepidemiological studies, veterinary pathology studies, experimental vector competence studies, ecological or transmission models, diagnostic studies, vaccine studies, systematic reviews or meta-analyses used for evidence mapping, narrative reviews used for background and citation tracing, and official risk assessments or guidance documents. Records were excluded when they were marginally relevant to RVFV, focused on unrelated pathogens without RVFV-specific inference, duplicated information available from stronger primary or official sources, lacked verifiable bibliographic or institutional metadata, or described endemic region observations without mechanistic, comparative, or operational relevance to the European preparedness question.

Records were independently assessed by two reviewers according to predefined eligibility criteria, and disagreements were resolved by discussion and consensus. Retained sources were checked for bibliographic identity through DOI/PMID, publisher, database, or official source records, relevance to the review question, and strength of inference. The final narrative synthesis retained 69 scientific and official institutional sources in the reference list.

The bibliographic screening pool comprised 2553 unique records; together with targeted official institutional sources, source selection yielded the 69 retained sources. Non-retained bibliographic records were excluded when one or more non-mutually exclusive criteria applied: insufficient RVFV-specific relevance to the review question; weaker duplication of evidence available from more direct primary or official sources; absence of verifiable bibliographic metadata or an interpretable English abstract, full text, publisher record, or official institutional record; endemic region observations without mechanistic, comparative, or operational relevance to European preparedness; or endpoints that did not support the inference required for the relevant synthesis section.

### 2.5. Data Extraction

For each retained source, extracted information included article or report type, year, country or region, host species or human population, vector species where applicable, sample size or denominator, diagnostic method, virological or serological endpoint, principal numerical findings, model output or scenario where applicable, vaccine or diagnostic relevance, surveillance or preparedness implication, European risk relevance, and major interpretive limitations. Quantitative values were extracted only when explicitly reported in verified scientific or official sources.

### 2.6. Evidence Appraisal and Handling of Conflicting Findings

Evidence was appraised through qualitative interpretive weighting; no formal study-level risk-of-bias score was applied. Weighting was based on how directly a source supported the preparedness question, the diagnostic endpoint used, the denominator and sampling context, the field or experimental setting, and the transparency of methods. Virus isolation, RT-PCR, RT-qPCR, and IgM evidence were treated as indicators of recent or active infection when the sampling context supported that inference. IgG and total-antibody results were interpreted as exposure evidence whose timing depended on assay, host population and denominator.

Evidence categories were interpreted according to the questions they answer. Vector competence studies informed biological plausibility; ecological models informed receptivity or scenario sensitivity; outbreak investigations informed clinical and operational impact; serological surveys informed exposure; and institutional risk assessments informed regulatory and preparedness context. Laboratory vector studies were compared through matched endpoints because mosquito species, colony or field origin, incubation temperature, infectious dose, feeding system, and definitions of infection, dissemination, and transmission can substantially affect interpretation.

Experimental vector competence, including infection, dissemination, and saliva or transmission endpoints, was interpreted as evidence of biological plausibility under defined laboratory conditions. Field detection of RVFV in vectors, vertebrate infection confirmed by virological methods, and epidemiological linkage between animal outbreaks and human occupational exposure were assigned greater interpretive weight than isolated vector presence, mosquito abundance, ecological suitability, or climate anomalies. Modeling studies contributed scenario-based evidence on receptivity, transmission potential, and preparedness sensitivity.

When findings differed across studies, the synthesis compared evidence by endpoint and context. Data from endemic African or Arabian Peninsula settings were retained when they clarified RVFV pathogenesis, vector–virus interaction, outbreak impact, diagnostic interpretation, vaccination, or surveillance failure modes. European, Mediterranean, and European Union-focused studies were prioritized when drawing conclusions about introduction pathways, receptivity, operational gaps, and proportional preparedness.

### 2.7. Narrative Synthesis

Evidence was synthesized thematically around RVFV etiology and pathogenesis, the animal–human interface, climate-sensitive emergence, vector expansion and vector–virus interaction, pathways toward Europe, surveillance, diagnostics, vaccination, biosecurity, and operational preparedness gaps. The synthesis distinguished evidence for viral circulation from evidence for exposure, vector competence, ecological suitability, introduction plausibility, and sustained transmission. Ethical approval was not required because the review used only previously published literature and official sources.

Consistent with a critical narrative design, sources were selected and appraised according to relevance, verifiability, endpoint directness, and interpretive strength. Conclusions were therefore framed as comparative and operational inferences across evidence categories, with pooled quantitative estimation outside the scope of this critical narrative design.

To make the appraisal process explicit, Table 1 summarizes how heterogeneous RVFV evidence categories were interpreted according to their endpoint, assumptions, limitations, and strength of inference for European preparedness.

## 3. Etiological Agent, Pathogenesis, and Animal–Human Interface

### 3.1. Viral Biology and Pathogenesis

RVFV is the etiological agent of RVF and belongs to the genus Phlebovirus within the family Phenuiviridae [1]. Its tripartite RNA genome links taxonomy to pathogenesis because viral polymerase activity, glycoprotein-mediated cell entry, nucleoprotein-supported replication, and non-structural proteins shape host–cell interaction, immune evasion, and virulence [1]. NSs is especially important: by suppressing host transcription and interferon responses, it allows viral replication before innate antiviral control is fully established [19,20]. This mechanism has epidemiological consequences, explaining acute viraemia, rapid tissue invasion, severe disease in susceptible animals and diagnostic difficulty during clinically silent or nonspecific infection.

Segment organization also matters for diagnostics and vaccines. The L, M, and S segments encode the RNA-dependent RNA polymerase, envelope glycoproteins and nucleoprotein/NSs, respectively [1]. These proteins determine replication, cell entry, immune suppression, and neutralizing antibody targets, linking viral biology to laboratory detection, attenuation strategies, and the interpretation of disease severity in immunologically naive livestock [19,20].

RVF pathogenesis varies across hosts and exposure contexts. In livestock, age and pregnancy status are particularly important because young animals and pregnant ruminants are most vulnerable to severe outcomes. Severe disease in neonates and abortion storms in sheep, goats, and cattle are among the most visible outbreak signals, and their interpretation should connect clinical appearance with mechanism. RVFV can infect placental tissues and the maternal–fetal interface, providing a biological explanation for fetal loss and for the high exposure risk generated by abortion materials during outbreaks [4,5]. Reproductive events are therefore central to RVF surveillance because they connect fetal pathology, herd-level amplification, and occupational exposure.

At the organ level, the key lesion linking RVFV pathogenesis with fatal disease is acute hepatocellular necrosis. In experimentally infected newborn lambs, liver lesions progressed rapidly from hepatocyte degeneration and necrosis within 6–12 h after inoculation to scattered necrotic foci by 12–24 h, larger circumscribed foci by 30–36 h, and massive hepatic necrosis by 48–53 h [21]. A later immunohistochemical study of experimentally infected newborn lambs and natural 1974–1975 field cases detected RVFV antigen most prominently in the liver, including cytoplasmic antigen in hepatocytes from 18 h post-infection and antigen in or adjacent to small hepatocellular necrotic foci at 24–33 h, supporting hepatocytes as a principal early site of viral replication [22].

This hepatotropic pattern connects pathology with epidemiology [1]. Rapid liver injury explains peracute deaths in neonatal lambs and kids, the seriousness of fetal and neonatal losses, and the central role of hepatic damage in severe systemic disease [1]. In humans, severe RVF may present as hepatitis and haemorrhagic fever [2,3]. Hepatic necrosis, thrombocytopenia, impaired coagulation, jaundice and elevated liver enzymes provide the biological bridge between systemic viral replication and haemorrhagic manifestations [2,3]. RVF pathogenesis is therefore best understood as a systemic hepatotropic disease with reproductive, vascular and neuro-ocular manifestations.

Human disease also has preventive relevance. Most recognized infections are febrile and self-limiting [2,3], whereas severe disease becomes clinically important when ocular, neurological, hepatic or haemorrhagic involvement develops. The preventive value lies in linking syndrome severity with exposure history, veterinary signals, relevant mosquito exposure and laboratory confirmation before severe human disease defines the event [7].

### 3.2. Animal–Human Interface, Sentinel Value, and Quantitative Evidence

Because pathogenesis and transmission are linked in infectious disease systems, RVFV transmission belongs within disease biology and epidemiology. The RVF-specific issue is how viral pathogenesis generates the conditions for population-level amplification. High-titer viraemia in susceptible ruminants can infect mosquitoes [1,23]. Placental and fetal infection can produce abortion materials with high occupational exposure potential [3,4]. Neonatal or herd-level disease can create visible animal health signals before or alongside recognized human cases [4,23]. Their sentinel value depends on whether surveillance captures them: animal signals are high-value detection and escalation triggers, while abortion or neonatal mortality clusters may already represent herd-level amplification and formal first recognition may still occur in humans where animal reporting is weak.

The animal–human interface is epidemiologically complex because animal amplification, vector transmission, and human exposure provide connected but distinct prevention targets. Viraemic livestock can infect mosquitoes and sustain amplification [3]. Recognized human infections often cluster around high-risk occupational contacts, with limited distribution across the general population [2,7]. This mixed transmission profile complicates prevention. Vector control addresses mosquito-mediated amplification, animal health control reduces viraemic–host availability, and occupational biosecurity reduces direct exposure to infectious animal materials [7].

Livestock sentinel value is central to preventive medicine because animal observations differ in timing and decision value. Compatible clinical disease, abortion clusters, and neonatal mortality are suspicion signals that should lead to farm investigation and laboratory sampling. Detection of IgM, RT-PCR-positive samples, or virus indicates recent or active infection and should escalate notification, animal sampling, and occupational exposure assessment. IgG alone has weaker temporal value, especially in endemic settings, and requires interpretation against the sampling frame, species, denominator and clinical context. Slaughterhouse sampling can add coverage where passive farm reporting is weak, but it becomes most informative when acute markers or spatially clustered animal events are present.

Thus, animal evidence should be graded by endpoint, timing and decision value. Severe reproductive losses during an outbreak provide stronger evidence of active epizootic transmission than isolated IgG seropositivity. Serological findings in clinically normal animals may indicate previous exposure, low-level circulation, or residual antibody from earlier transmission periods. For European preparedness, this grading links each signal to a proportionate response: compatible animal events justify field investigation, acute markers justify confirmatory testing and notification, clustered signals justify expanded sampling, and overlap with seasonal receptivity justifies vector or environmental assessment.

**Figure 1:**
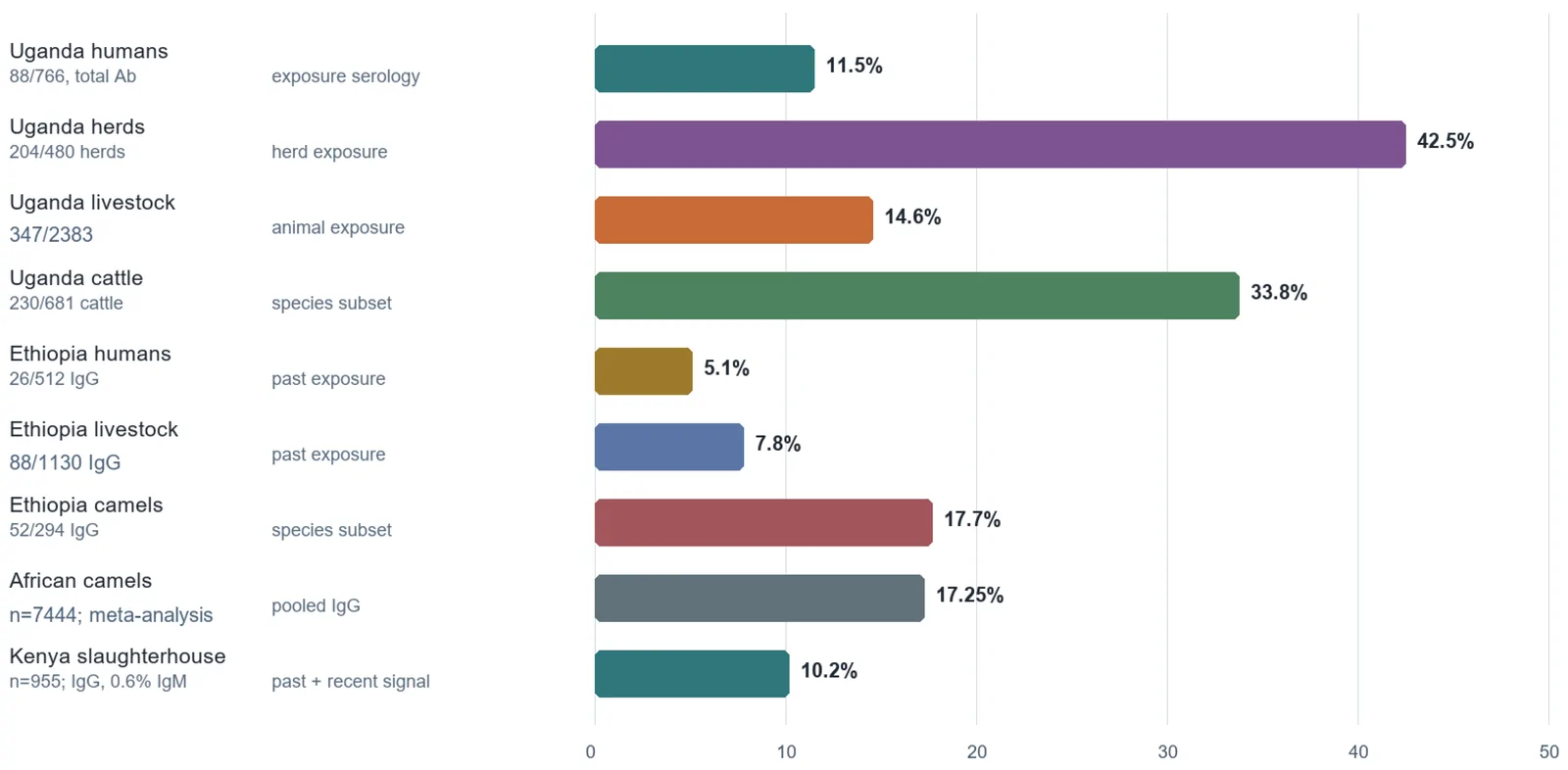
Selected RVFV serological exposure estimates with numerator/denominator or sample size information and interpretive category. Most estimates represent IgG or total-antibody evidence of past exposure from heterogeneous One Health and livestock serological studies [24,25]. Camel serology is shown separately because its interpretation depends on mobile–livestock interfaces [26]. The Kenya slaughterhouse study additionally reported 0.6% IgM positivity, suggesting recent infection in a small subset [27]. Values are source-specific examples; pooled regional prevalence or active infection interpretation would require harmonized sampling and acute infection markers. The figure supports endpoint interpretation for surveillance decisions; European prevalence and outbreak probability are assessed through European pathway evidence, local receptivity, and virological confirmation.

Endemic region evidence is retained here only to calibrate endpoints that a European preparedness system might need to interpret after a credible RVFV alert; European prevalence, trade exposure and outbreak probability are assessed through pathway, receptivity, virological confirmation, and transmission modeling evidence. The Uganda and Ethiopia surveys show that IgG results in people, herds, individual animals and species groups do not carry the same decision value. Uganda reported 11.5% (88/766) human seropositivity, 42.5% (204/480) herd-level seropositivity, 14.6% (347/2383) animal-level seropositivity, and 33.8% (230/681) cattle seropositivity [24]. Ethiopia reported 5.1% (26/512) human IgG, 7.8% (88/1130) livestock IgG, and 17.7% (52/294) camel IgG [25]. Figure 1 presents these estimates with denominators and IgG/IgM interpretation, preserving the distinction between exposure evidence and acute infection signals.

Risk-factor findings from these surveys identify failure points after animal infection has occurred. In Uganda, raw meat consumption was associated with human RVFV seropositivity, with an adjusted odds ratio of 6.11, and lack of carcass burial was associated with herd-level seropositivity, with an adjusted odds ratio of 15.70, although confidence intervals were wide [24]. In Ethiopia, handling aborted materials and recent unusual mosquito abundance were associated with human RVFV IgG seropositivity [25]. For Europe, the transferable lesson is operational rather than prevalence-based: suspicion should lead first to animal event investigation and safe management of carcasses or abortion material, while occupational protection and vector–context assessment define the immediate exposure perimeter.

Camel serology carries limited and indirect evidentiary weight for continental European preparedness. A systematic review and meta-analysis reported a pooled RVFV seroprevalence of 17.25% among 7444 African dromedary camels from 34 studies and 11 countries, with substantial heterogeneity [26]. Its interpretive value is restricted to movement interface interpretation: serological exposure in mobile livestock systems can mark interfaces connecting East Africa, North Africa, and the Middle East, where animal movement and trade ecology differ from European Union import controls [11,26]. For the European situation, this evidence is useful mainly for separating non-European movement interface vulnerability from continental European risk assessment, which depends more directly on regulated entry routes, local vector–host receptivity, and virological confirmation.

Two recent non-European operational studies are retained because they mark different decision endpoints. Southern Kenya slaughterhouse surveillance found 10.2% IgG positivity among 955 livestock, a rise to 22.6% by May 2024, and 0.6% IgM positivity without visible lesions, illustrating how acute markers in abattoir sampling may reveal infection missed by passive clinical reporting [27]. The Rwanda outbreak investigation confirmed 28 RVFV-positive animals among 4062 sampled, reported 10.7% case fatality among positives, vaccinated 112,110 animals, and contained the outbreak within 51 days without reported human cases [28]. These studies calibrate the difference between detection through surveillance and response after confirmed outbreak; European trade risk and outbreak probability are addressed through European pathway, receptivity, and institutional risk evidence.

These examples support endpoint-based interpretation rather than direct geographic extrapolation. Cross-sectional surveys calibrate serological endpoints [24,25], camel evidence illustrates mobile–livestock interface uncertainty [26], slaughterhouse surveillance shows the value of acute markers [27], and outbreak investigation links confirmation with vaccination and reporting speed [28]. In this framework, European circulation, sustained transmission and outbreak probability are inferred from European and Mediterranean entry routes, local receptivity, virological confirmation and transmission modeling.

## 4. Climate-Driven Emergence and Environmental Suitability

### 4.1. Climate, Hydrology, and Amplification Conditions

Climate-driven emergence in RVF is best interpreted as an ordered amplification process. Weather anomalies create opportunity, but viral introduction, competent vectors, and susceptible hosts determine whether amplification follows. Rainfall and flooding create mosquito habitat in livestock disease and RVF outbreak analyses [12,13]. Environmental suitability modeling identifies surveillance windows [14], while reanalysis of the Southwestern Arabian outbreak shows that wet conditions became important only in the context of introduction history [15]. Vector transmission also depends on adult mosquito survival and temperature-dependent extrinsic incubation [29]. Host–vector contact gains epidemic relevance when a viral source reaches susceptible ruminants, as emphasized by global health syntheses and research priority work [16,17], and by interepidemic modeling [18]. For Europe, climate and environmental monitoring should therefore focus investigation after credible animal, vector, movement or clinical signals.

Evidence from endemic and epidemic regions shows that environmental suitability interacts with animal movement and human behavior. In Madagascar, environmental conditions modulated local RVFV transmission in ruminants, but ruminant trade and cattle movement between trade hubs were identified as major drivers of long-distance spread; contact with cattle from infected districts was associated with higher infection risk in slaughterhouse workers [13]. This challenges climate-only explanations of RVF emergence and is directly relevant to Europe, where environmental suitability must be evaluated together with movement pathways.

The 2000–2001 outbreak in Southwestern Arabia provides a complementary lesson. RVFV was considered to have been introduced into the Arabian Peninsula during or after the 1997–1998 East African outbreak and before August 2000, possibly through wind-blown infected mosquitoes or infected animals. A subsequent wet period produced abundant amplification vectors, and the outbreak resulted in more than 1500 diagnosed human cases and at least 215 deaths. However, favorable environmental conditions later occurred without subsequent outbreaks, indicating that rainfall and inundation alone were insufficient for repeated emergence [15].

### 4.2. Environmental Modeling and European Interpretive Limits

Modeled environmental suitability is useful for preparedness but must be interpreted by endpoint. A large suitability analysis used RVF occurrence data and animal notifications to generate monthly predictions from January 1995 to December 2016 at 5 × 5 km resolution across Africa, Europe, and the Middle East. The model included 1381 reports from 32 countries and correctly classified 97.8% of point cases as suitable in the month and year of occurrence [14]. Such models can identify where surveillance may be more efficient, while confirmation of transmission still depends on virological, serological, animal health, entomological, or outbreak investigation evidence.

East African global-change modeling is retained to show how climate-sensitive RVF risk can behave non-linearly. The model identified interepidemic hotspots in Eastern Kenya, Eastern Tanzania, and Southwestern Uganda, with hydrology as a major driver and May–July as the peak risk period after the long rains. Although projected disease risk generally decreased under three global-change scenarios, population growth increased the estimated number of exposed people from about 49 million historically to more than 90 million by 2061–2080 [18]. For Europe, the value of this evidence is methodological: environmental suitability, livestock distribution and population exposure can change in different directions.

Across the climate and modeling literature, evidence must be separated by scale and endpoint. Outbreak investigations reconstruct introduction amplification sequences [13,15]. Suitability models identify permissive space–time windows [14]. Global health and research priority syntheses define prevention needs beyond outbreak description [16,17]. Global-change models estimate future exposure and uncertainty under changing demography, hydrology, livestock distribution, and land use [18]. Their agreement supports climate-sensitive preparedness, while their different endpoints and causal resolution prevent deterministic climate emergence claims.

## 5. Mosquito Vectors and Transmission Plausibility at Europe’s Risk Frontier

### 5.1. Vector Competence, Local Receptivity, and Endpoint Weighting

Available European and Mediterranean vector evidence supports geographically and seasonally bounded transmission plausibility. Experimental competence becomes operationally relevant only when a plausible entry event reaches viraemic livestock, sufficient vector abundance, and susceptible ruminants in the same place and season. The Mediterranean meta-analysis provides comparative endpoint estimates across candidate vectors [30], while Spanish and Dutch experiments give more direct evidence for local mosquito populations and a European livestock–vector interface [31,32]. EU risk assessment and mosquito ecology sensitivity analysis then define where these endpoints matter for mapping, targeted trapping or bounded RVFV testing [11,33]. Vector–maintenance synthesis addresses the separate question of whether mosquito–virus interactions could support persistence between recognized outbreaks [34].

The first level of evidence is within the mosquito itself. RVFV survival between recognized epidemics depends on mosquito–virus interaction as well as vertebrate amplification. After a mosquito feeds on a viraemic host, RVFV must overcome within-vector barriers, replicate, disseminate, and either reach saliva for horizontal transmission or persist through reproductive pathways that may seed future mosquito cohorts. The vertical/transovarial transmission hypothesis is therefore central to inter-epizootic maintenance: infected eggs may survive environmentally unfavorable periods and later hatch into infected adults, creating a mechanism for viral persistence before overt livestock disease is detected. Lumley et al. synthesized this maintenance framework and emphasized that long-term survival depends on vertical transmission to mosquito progeny, egg-associated survival, and subsequent amplification in competent vectors and susceptible vertebrate hosts [34].

Evidence for this maintenance pathway is biologically plausible but uneven. Stochastic host–vector modeling by Pedro et al. linked interepidemic RVF activity to transovarial transmission by *Aedes mcintoshi* and showed that vertical transmission changes invasion and extinction probabilities, meaning that persistence is a dynamic vector–host process [35]. Manore and Beechler [36] used a between-season persistence model and showed that persistence depended jointly on vertical transmission rate, vector-to-host ratio, host availability, and alternate competent hosts; in that model, reducing the minimum vector-to-host ratio below 20:1 required approximately 3% consistent vertical transmission and at least 1000 buffalo, whereas alternate hosts reduced persistence thresholds [36]. Bergren et al. then provided species-specific laboratory evidence by detecting infectious RVFV in progeny from *Culex tarsalis* across three successive gonotrophic cycles after oral exposure, with progeny infection rates of 2.0–10.0% and low viral titres [37].

For European risk assessment, these findings provide biological plausibility with species-specific uncertainty. Vertical transmission and persistence evidence is uneven across candidate vector species [34,37]. Findings from *Aedes mcintoshi*, *Aedes vexans*, or *Culex tarsalis* therefore require cautious interpretation before they are applied to European *Culex pipiens*, *Aedes caspius*, *Culex theileri*, or *Stegomyia albopicta* populations. Species-specific field and laboratory data remain necessary for European inference, particularly where EU risk assessment and European mosquito experiments identify local competence or receptivity questions [11,31].

The Netherlands and Spanish experiments provide the most directly relevant European mosquito data in this section because they test local vector populations or European livestock–vector interfaces. In the Netherlands lamb-to-mosquito experiment, artificial feeding produced dose-related infection rates of 30–74% and transmission rates of 8–24%, while feeding on viraemic European-breed lambs produced infection rates of 86–91% and transmission rates of 29–30% during peak viraemia, with much lower values outside that narrow window [32]. In Spain, local populations of *Culex pipiens* hybrid form and *Stegomyia albopicta* supported dissemination and infectious virus in saliva after exposure to a virulent RVFV strain [31]. Together, these studies support conditional vector-mediated transmission plausibility in Europe: mosquito infection after exposure and infectious virus in saliva show that some local populations can reach late competence endpoints, and the lamb-feeding experiment links peak livestock viraemia with mosquito infection [31,32]. The magnitude of field risk would depend on introduction timing, viraemic livestock, vector density, seasonal temperature, and speed of animal health detection, consistent with the conditional interpretation used in EU risk assessment [11].

### 5.2. Comparative Vector Evidence and Surveillance Implications

The Mediterranean vector competence meta-analysis provides a broader comparative perspective. Among major potential RVFV vectors considered in the Mediterranean Basin, *Aedes caspius* had an infection rate estimate of 96.7%, with a 95% confidence interval of 77.9–100%, but its overall transmission rate was only 9.8%, with a 95% confidence interval of 7.1–12.9%. *Culex pipiens* had an infection rate estimate of 68.0% and an overall dissemination rate of 13.5%, while *Culex theileri* had a high infection rate estimate of 88.6% but lacked downstream transmission estimates [30]. The comparison is useful because the endpoints represent different biological steps in vector competence: infection reflects susceptibility after exposure, dissemination reflects spread beyond the midgut, and transmission requires infectious virus in saliva or successful passage in host-to-vector or vector-to-host experimental systems.

**Figure 2:**
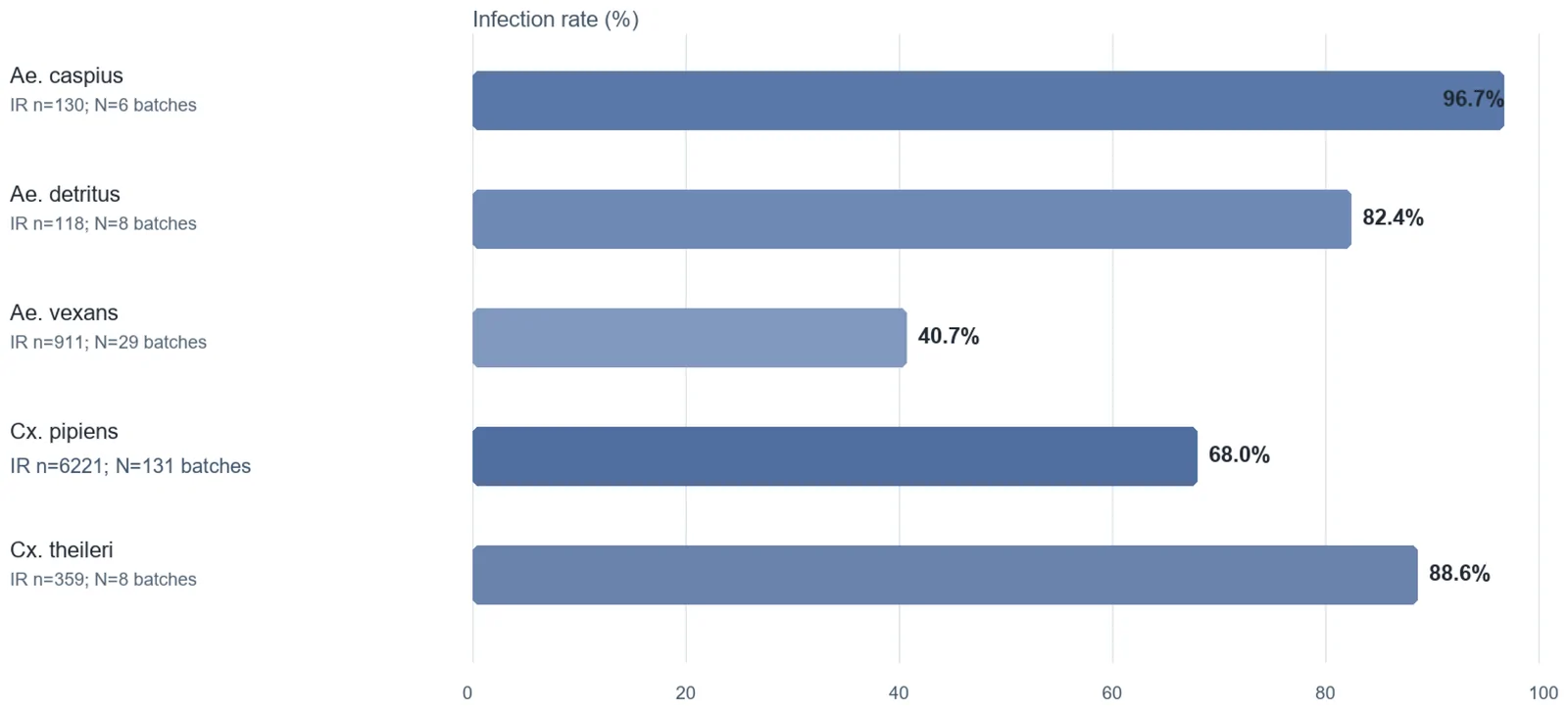
Infection rate estimates for Mediterranean mosquito vectors. Point estimates are derived from a vector competence meta-analysis and represent experimental infection rates under defined laboratory conditions [30]. Denominators are shown as the number of mosquitoes (*n*) and experimental batches (N) included in the source meta-analysis: *Aedes caspius* *n* = 130/*N* = 6, *Aedes detritus* *n* = 118/*N* = 8, *Aedes vexans* *n* = 911/*N* = 29, *Culex pipiens* *n* = 6221/*N* = 131, and *Culex theileri* *n* = 359/*N* = 8. These values represent mosquito infection after experimental exposure and are therefore a susceptibility endpoint. Stronger inference for European transmission plausibility requires additional endpoints, including dissemination, infectious virus in saliva, host-to-vector or vector-to-host transmission experiments, and, in the field, virological detection or epidemiologically linked outbreak evidence. Because experimental conditions differ among source studies, the figure is limited to laboratory susceptibility comparison; field transmission requires virological or outbreak confirmation.

For preparedness, mosquito endpoints should be weighted by proximity to onward transmission. Infection after exposure demonstrates susceptibility. Dissemination shows movement beyond the midgut. Infectious virus in saliva or host-to-vector/vector-to-host passage gives stronger laboratory support for amplification during a receptive season [30]. European experiments therefore add more than presence data. They show late competence endpoints in local mosquito populations and efficient mosquito infection during peak lamb viraemia [31,32]. This evidence supports targeted escalation when a plausible entry or animal health signal occurs in a receptive area. Baseline mapping remains appropriate where competent or potentially competent vectors coexist with susceptible livestock [11]. Figure 2 presents Mediterranean candidate–vector infection estimates by species and denominator within this endpoint-weighted interpretation.

For European preparedness, vector surveillance is most informative when it is tied to a decision point. Baseline mapping can identify locations where competent or potentially competent mosquitoes coincide with dense susceptible livestock, wetlands or irrigated habitats, ports, airports, or other points of entry [11,30]. After a movement alert, compatible livestock event, laboratory signal, or unusual seasonal vector abundance, European vector competence and host-to-vector evidence can support targeted trapping, species identification, and locally bounded RVFV testing [31,32].

Entomological preparedness should turn baseline vector information into surveillance thresholds. Repeated mapping identifies receptive zones when competent or suspected vectors occur near susceptible livestock during suitable seasons [11,30]. Routine awareness and trapping capacity can be maintained in those zones. RVFV testing of mosquito pools belongs to a higher decision tier, activated by credible entry, compatible animal disease, laboratory evidence, occupational exposure, or unusual vector activity. This structure gives vector mapping a defined preparedness role and links molecular testing to an epidemiological signal.

## 6. Pathways Toward Europe and Preparedness Gaps

### 6.1. Entry Pathways, European Receptivity, and Conditional Establishment

European entry-route analysis should ask which pathways can deliver viable RVFV to settings where livestock amplification could occur. Viraemic animals and infected transported vectors are the most direct biological mechanisms. Human travel, environmental suitability and vector presence have a different use: they inform differential diagnosis, receptivity mapping and surveillance timing. European analyses identify virus-carrying vectors and viraemic hosts as plausible pathways [9,10], and broader arbovirus-emergence work supports animal trade and migration as transboundary mechanisms [38]. In Mediterranean interfaces, permissive environmental windows may overlap locally with competent mosquitoes and susceptible ruminants [14,30]. The limiting operational question is whether an entry event reaches livestock–vector contact during that window before detection.

Live-animal movement is biologically direct because viraemic ruminants or camels can connect viral circulation in endemic or epidemic areas with susceptible animals, competent vectors, and occupational exposure in receiving regions. EFSA identified movement of live animals as the most important pathway for RVF spread from African endemic areas to North Africa and the Middle East, and noted that Horn of Africa trade routes toward the Arabian Peninsula were implicated in the 2000 Saudi Arabia and Yemen introduction. This pathway is therefore a more appropriate comparator for RVF than malaria, dengue, or chikungunya travel surveillance. Under strict European Union (EU) animal import controls, EFSA estimated the risk of EU introduction through infected animals as very low, while residual concern remains greatest for neighboring regions, informal movements, and risk–frontier interfaces where controls may be less uniform [11].

EFSA’s 2026 pathway identification report adds a recent EU-level update by distinguishing potential RVFV entry routes into currently free Member States. The report identifies aircraft-transported mosquitoes, movement of cattle, sheep and goats, germinal products and selected animal products as possible routes; the latter include raw milk, fresh meat and contaminated hides or skins. Its scope is pathway identification, so the routes require further interpretation by regulatory context and establishment potential. EU Member States, neighboring European or Mediterranean regions and European overseas territories should be considered separately because their animal–movement interfaces, baseline epidemiology, and surveillance capacity differ [39].

Mayotte is discussed only as a geographically distinct cautionary example from the Southwestern Indian Ocean. The 2018–2019 outbreak occurred in a French overseas department embedded in a Comorian/East African animal movement and public health context. It involved 142 confirmed human cases, 73% reported animal or biological fluid exposure, an increase in cattle seroprevalence from 3.6% to 10.1%, and 165 animal polymerase chain reaction (PCR)-positive cases [40]. Mosquito data were limited to predominance of *Culex*, without reported RVFV testing of mosquito pools. The example illustrates signal integration, delayed animal detection, occupational exposure, and the need to interpret overseas territory events within their regional animal health and public health context.

Vector transport represents a different pathway. EFSA estimated the overall risk of RVF introduction into the EU through infected vectors as very low, with the highest estimates for Belgium, Greece, Malta, and the Netherlands, mainly linked to air and sea connections with African RVF-infected countries. Country-level Method for INTegrated RISK Assessment (MINTRISK) outputs also showed heterogeneity: Bulgaria had an overall vector pathway introduction score of −0.52, while the Netherlands and Greece had higher point estimates of 0.33 and 0.23, with wide uncertainty intervals [11]. The findings support model-based pathway prioritization and differentiated European risk framing across points of entry and receptive areas.

Human travel is considered to pose a negligible risk of onward RVFV transmission in Europe because recognized human infection is often occupationally linked [2,3]. Disease prevention evidence identifies exposure history as the relevant clinical and public health context for interpreting travel-associated suspicion [7]. EFSA assessed human travel as a negligible pathway for RVFV introduction [11], and traveler surveillance data support its main value as a differential diagnostic and sentinel awareness signal [41]. Table 2 summarizes pathway-specific and preparedness-layer interpretations for this and the other evidence categories.

Receptivity provides a useful concept for separating exposure pathways from local transmission potential. Exposure pathways describe opportunities for viral entry, whereas receptivity describes whether local vectors, hosts, seasonality, and environmental conditions could support amplification after entry. Spatial risk analysis in the Netherlands mapped ecological conditions for the introduction and establishment of six arboviruses, including RVFV, and identified clustered high-hazard areas, especially in the southern part of the country [42]. Its contribution is operational: it indicates where multiplex surveillance may be more efficient when vector abundance, host availability, abiotic conditions, and introduction signals overlap.

Spatial risk mapping contributes one layer of evidence, whereas dynamic transmission modeling adds timing and intervention sensitivity. Fischer et al. [43] developed a deterministic RVFV model for 5 km by 5 km Dutch grid cells using livestock abundance, vector abundance, temperature-dependent vector parameters, infected mosquito eggs, initial growth rate, and seasonal persistence. The main result was non-linear: seasonal persistence depended on vector-to-host ratios, so areas with lower livestock density could have higher persistence potential than densely populated livestock areas [43]. EFSA then added the response dimension. In a continental EU scenario with R0 of 2, mean vector dispersal of 10 km and 10 initially detected farms, spread beyond 100 km had a 10% probability, and spread beyond 50 km had a 55% probability. Culling within 20 km was modeled as effective but highly disruptive, whereas ring vaccination within 20–50 km emerged as an alternative [44]. These studies are interpreted as transmission dynamics evidence, separate from spatial suitability and vector competence evidence.

Dynamic modeling converts receptivity from a mapped condition into a response problem. Dutch modeling and EFSA scenarios show that vector-to-host ratios, livestock density, temperature-dependent vector parameters, surveillance sensitivity, dispersal assumptions, and acceptable control measures change whether an incursion remains limited or becomes livestock amplification [43,44]. Where local receptivity and plausible entry overlap, preparedness should move from background awareness to conditional prioritization. The operational assessment identifies specific place–season combinations that justify targeted clinical awareness, abortion and neonatal mortality investigation, pathway review and rapid diagnostic escalation [45,46].

European experience with other vector-borne zoonoses supports this consequence-weighted interpretation. West Nile virus lineage 2 was introduced into Europe and spread across several European settings [47], and autochthonous Crimean-Congo haemorrhagic fever in Spain showed that vector-associated zoonotic viruses can emerge in European Union contexts when pathogen presence, competent vectors, exposed hosts, and delayed recognition coincide [48]. These examples strengthen the realism of an RVFV incursion scenario as preparedness comparators, while RVFV probability requires its own livestock-vector evidence. RVFV epidemic amplification would depend on viraemic livestock, competent mosquitoes, and susceptible ruminant populations. This distinguishes RVFV from the bird–mosquito cycle of West Nile virus and from the tick-borne livestock–human interface of Crimean-Congo haemorrhagic fever. The operational lesson for RVF preparedness is that rare but high-consequence vector-borne introductions require targeted surveillance thresholds and response planning before clinical amplification becomes extensive.

Table 2 integrates entry-route evidence with preparedness evidence layers used to interpret whether an introduction pathway could become operationally important. It supports the first step of the conditional convergence framework by separating direct entry mechanisms from receptivity indicators, modeling evidence, and context-specific preventive medicine implications.

**Table 2:** Evidence-based interpretation of selected RVFV entry routes and preparedness evidence layers at Europe’s risk frontier.

Entry Route or Preparedness Evidence Layer	Quantitative or Empirical Signal	Interpretive Limit	Preventive Medicine Implication
Live-animal movement	Main pathway from African endemic areas to North Africa and the Middle East; less than one EU epidemic every 500 years via infected animals under strict EU import policy [11].	Very-low-risk EU estimate under regulated entry does not generalize to Middle Eastern or informal movement interfaces.	Maintain regulated import controls and passive abortion/mortality reporting; escalate to laboratory investigation only when animal health, movement, or import alerts occur.
Infected-vector transport	Highest EU vector pathway risk: one epidemic every 228–700 years for Belgium, Greece, Malta, and the Netherlands [11].	Modeled introduction estimate for pathway prioritization under specified assumptions.	Maintain targeted vector mapping at points of entry and receptive areas; apply RVFV pool testing during signal-triggered periods, with routine broad screening reserved for settings of higher expected yield.
Germinal products and selected animal products	EFSA 2026 identifies RVFV germinal products and selected animal products, including raw milk, fresh meat, and contaminated hides or skins, as potential entry routes, alongside cattle, sheep and goat movement and aircraft-transported mosquitoes [39].	Pathway identification requires subsequent ranking by viability, exposure, regulatory control, and establishment conditions.	Maintain regulated controls for germinal products and animal products, import documentation, border awareness, and event-triggered tracing; avoid treating product pathways as equivalent to viraemic live-animal introduction.
Southwestern Indian Ocean/French overseas outbreak context	Linked human, cattle-serological, and animal PCR signals occurred under French jurisdiction in a Southwestern Indian Ocean setting [40].	Regional animal movement, public health, and animal health context differs from continental Europe; reported mosquito data describe genus composition, without RVFV pool-testing evidence.	Use as a cautionary signal integration example for coordinated animal–human investigation and occupational protection.
Human travel surveillance	Traveler surveillance identified imported RVF rarely relative to malaria, dengue, and chikungunya [41].	Traveler surveillance has limited value as an establishment–pathway comparator because RVF is occupationally linked and requires livestock–vector amplification, unlike predominantly human–vector–human travel-associated comparators.	Use travel history for differential diagnosis and sentinel awareness, not as a reason for active population-level RVF surveillance.
Preparedness evidence layer: local ecological receptivity	Netherlands arbovirus mapping identified clustered high-hazard areas, especially in the south [42].	Hazard maps indicate suitability and help define where escalation would be most efficient after a credible signal.	Use maps to predefine signal-triggered enhanced surveillance areas after introduction, animal, vector, or environmental triggers.
Preparedness evidence layer: dynamic transmission modeling	Dutch dynamic modeling and EFSA control scenarios link spread potential to seasonality, vector-to-host ratio, introduction timing, dispersal assumptions, and intervention choices [45,46].	Scenario-dependent evidence; model outputs estimate spread and intervention sensitivity under specified assumptions.	Use models to predefine escalation thresholds, zoning, and vaccination/culling scenarios; apply enhanced surveillance after detection or credible incursion signals.

### 6.2. Surveillance, Diagnostics, Vaccination, and Operational Gaps

European RVF surveillance should be risk-based because severe human disease would be a late and insensitive trigger for an animal-amplified zoonosis. EFSA concluded that passive surveillance based on notified ruminant abortions is currently the only feasible animal health warning basis in the EU [44]. Enhanced surveillance becomes more appropriate when introduction risk rises around possible incursion points during vector-active periods [44]. In disease-free settings, abortion or neonatal mortality notifications may be the first practical signal available to authorities. Biologically, however, they may already indicate herd-level amplification. Their value is to start farm investigation, laboratory confirmation and occupational-risk reduction before wider animal or human involvement is recognized. Escalation should depend on signal quality, geographic clustering and supporting movement, vector, environmental or laboratory evidence.

In EU Member States, RVF category A status means that suspicion or confirmation activates competent authority action, including risk-based sampling, zoning and time-bound control measures [45]. Non-EU European countries should similarly define the national notifiable-disease route, responsible veterinary authority, reference-laboratory pathway and cross-border communication channel before a suspect event occurs. This legal status creates a resource allocation problem because RVFV preparedness competes with surveillance for other livestock diseases [49,50]. Surveillance sensitivity will vary with livestock density, producer and practitioner reporting, and differential abortion diagnostics [46]. It also depends on sample transport and laboratory interpretation [51,52]. Regional One Health coordination adds another source of variation [53,54]. Delayed abortion reporting or diagnostic work-up would weaken passive surveillance in the EU context described by EFSA [44].

Operationally, RVFV should enter syndromic abortion and neonatal mortality investigation as a trigger-dependent differential diagnosis. Ruminant abortion diagnosis requires coordinated input from producers, practitioners and diagnosticians, and many investigations remain unresolved even after systematic sampling [46]. Farm veterinarians, slaughterhouse services and diagnostic laboratories should add RVFV PCR and IgM/IgG testing when compatible animal events coincide with movement or product-entry concern, vector-active conditions, occupational exposure, or other convergent signals [51,52]. Syndromic surveillance evidence supports this approach because it links nonspecific animal health signals with structured escalation [49,50].

The allocation rule is therefore to strengthen shared livestock surveillance infrastructure at baseline and reserve RVFV-specific expansion for convergent signals. Baseline resources should maintain veterinary practitioner awareness that RVFV belongs in the differential diagnosis of abortion or neonatal mortality when epidemiological context supports suspicion, while also supporting routine reporting, differential sampling, sample transport, and laboratory access for multiple livestock diseases [46,49]. RVFV-specific PCR and IgM/IgG testing should be added when that shared syndromic system detects compatible disease in a receptive setting with movement, product-entry, vector-active, occupational exposure, or laboratory context [51,52]. This allocation model embeds RVFV in routine syndromic livestock surveillance while preserving a clear route to rapid competent authority escalation when evidence supports it [45].

European and Mediterranean surveillance assessments support this integrated framing. Analyses of West Nile virus and RVF surveillance in European Mediterranean countries, together with entomological harmonization guidance, show that surveillance objectives must be context-specific and connected across human, veterinary, entomological and environmental systems [53,54]. Broader European mosquito-borne-disease surveillance supports the same coordination principle for vector monitoring within integrated surveillance systems [55].

Diagnostic readiness is equally important because RVF clinical signs are nonspecific in animals and humans. Molecular testing is most useful during acute viraemia, while IgM and IgG serology answer different questions about recent infection and previous exposure. Diagnostic algorithms must therefore be designed around the surveillance question: suspected acute human disease, livestock abortion cluster, animal import investigation, vector-pool screening, slaughterhouse sentinel sampling, or retrospective exposure assessment [51,52].

Point-of-care or near-patient diagnostics may shorten time to action, but they require field validation. A systematic review and meta-analysis of rapid and near-patient diagnostics for RVF and Crimean-Congo haemorrhagic fever included 17 studies, of which 14 entered meta-analysis. RVFV diagnostics showed consistently high accuracy, but most evaluations occurred outside endemic regions and real-world field validation remained limited [52]. Operational readiness also requires biosafety, sample transport, confirmatory laboratories, differential diagnostic panels, and communication routes between veterinary and public health laboratories.

In European RVF preparedness, vaccination should be defined before an event as a conditional emergency control option for livestock amplification. Under routine conditions, the appropriate action is regulatory and logistical readiness because current EU assessments place entry and establishment at very low baseline probability [11]. If RVFV infection is confirmed or strongly suspected during vector-active conditions in susceptible livestock, the control question changes from planning to deployment. EFSA control modeling indicates that ring vaccination can be an alternative to highly disruptive culling under specified outbreak scenarios [44]. Regulatory readiness therefore requires a verified product route through the EMA Union Product Database and an emergency-use plan under Commission Delegated Regulation (EU) 2023/361 [56,57]. That plan should specify the competent authority, geographic ring or target zone, eligible species, movement conditions for vaccinated animals and products, and reinforced surveillance for reproductive and neonatal losses.

Product selection should be ranked by legal deployability, protection in the target species, safety in pregnant animals, and compatibility with post-vaccination surveillance. Vaccine development evidence supports livestock vaccination as a plausible control approach, but preparedness value differs by product and field constraint [58]. Pregnancy safety carries particular weight because RVF outbreak impact is dominated by fetal death, neonatal mortality, and infectious abortion materials. Historical first-trimester exposure to RVF and Wesselsbron vaccines was associated with severe fetal and placental outcomes [59], MP-12 produced abortions and teratogenic effects in pregnant sheep [60], and Clone 13 crossed the ovine placental barrier under experimental overdose conditions [61]. These data place pregnancy status at the center of emergency-use restrictions before mixed-pregnancy flocks or herds are considered.

Candidate and deployable platforms should be interpreted by evidence strength. Clone 13 has live-attenuated efficacy evidence in sheep [62], but placental-passage findings constrain its preparedness role [61]. ChAdOx1 RVF adds pregnant sheep and goat protection data [63]. Four-segmented RVFV addresses prevention of vertical transmission in pregnant ewes [64], and NSs/NSm-deleted vaccine evidence supports attenuation, protection and pregnancy safety [65]. Human ChAdOx1 RVF phase 1 data show adult tolerability and immunogenicity. Veterinary deployment still depends on product authorization, livestock efficacy, pregnancy safety evidence, field availability and DIVA-compatible surveillance [66].

Implementation capacity should be assessed after product suitability. A suitable vaccine has little control value if procurement, release or cold-chain distribution cannot meet the short incursion window. DIVA-compatible monitoring should be fixed before deployment so that post-vaccination serology can distinguish new infection from vaccine-associated responses [51,52]. Emergency-vaccination rules shape product use, movement restrictions and post-vaccination monitoring [56,57]. Vaccine bank access, stockpiling arrangements, procurement time, shelf life and field-team capacity remain lower-certainty but operationally decisive issues [67,68]. One Health exercises can test the full delivery pathway before an event [69]. The preparedness position is therefore consistent: surveillance detects and localizes the event, while vaccination becomes a livestock-control option when infection is confirmed or strongly suspected.

Biosecurity and occupational protection should follow the main human exposure route: direct contact with infectious animal materials. Routine meat inspection, milk hygiene, slaughterhouse controls and carcass disposal already reduce several zoonotic risks and would also reduce RVFV exposure after an incursion. The RVF-specific risk emerges during an undetected animal event, when veterinarians, farmers or slaughterhouse workers may handle fresh blood, fetal membranes, aborted fetuses or carcasses from viraemic animals. Preparedness should keep standard food safety and slaughter-hygiene practice as the baseline. Event-specific control should then focus on unsafe slaughter or carcass movement, safe disposal of abortion material, personal protective equipment, farm-level communication and rapid laboratory investigation [24,25].

Operational preparedness depends on linking each signal to an action threshold. Routine periods require professional awareness, laboratory access, pathway mapping and seasonal vector information. Compatible livestock disease or credible movement concern should trigger targeted investigation, especially when occupational suspicion or concentrated receptivity is present. RVFV IgM, PCR detection or convergent multisector evidence should trigger emergency response, with EFSA-supported notification, trace-back, zoning and movement control when legally and epidemiologically justified [44]. Diagnostic algorithms clarify whether acute infection, previous exposure or discordant serology is being interpreted [51,52], and vaccination or culling should follow pre-agreed legal and operational pathways [67].

Table 3 operationalizes the framework by mapping six European risk frontier scenarios to their warning signal, responsible authority, immediate response with spatial expansion, and underlying evidence. This mapping shows how decision thresholds differ across evidence types: an indirect signal supports readiness or investigation, whereas direct virological or multisector evidence supports emergency response.

Research priorities should be selected by their capacity to reduce uncertainty at defined decision points. Outbreak-control modeling can evaluate trigger thresholds and the likely benefit of alternative spatial responses [44], while vector competence experiments at realistic seasonal temperatures can narrow the period in which local amplification is biologically plausible [30]. Diagnostic studies are needed to distinguish acute infection from previous exposure [51,52], and operational studies should measure whether veterinary reporting, laboratory confirmation and public health notification connect quickly enough for intervention [49,50]. Entomological work has greatest preparedness value when it is linked to animal health surveillance [53,54] and Mediterranean mosquito-borne coordination [55]. Vaccine-readiness research should address legal access, stockpiling and field delivery within the short incursion window [67,68], with One Health exercises testing activation before an event [69]. Together, these priorities translate research into decisions about when to investigate, where to expand surveillance and when control becomes proportionate.

## 7. Limitations

The evidence base is heterogeneous, so inferential strength varies. Continental Europe still lacks direct field evidence of autochthonous RVFV circulation. Preparedness inferences therefore rely on mechanistic studies, Mediterranean and neighboring-region context, and operational lessons from endemic settings. Serological interpretation depends on assay and denominator as much as on percentage. Model outputs depend on vector–host assumptions and intervention timing. Recommendations are also constrained by legal authorization, diagnostic capacity, veterinary reporting and resources shared with other livestock diseases. These limitations support proportionate, trigger-based preparedness within a defined European risk context.

## 8. Conclusions

RVF should be understood as an etiologically defined viral zoonosis whose European relevance lies in preparedness for a rare but potentially consequential incursion. Current evidence supports low-probability risk under existing EU conditions [11], while earlier European risk and public health analyses explain why the disease remains relevant for preparedness despite absence of endemic continental transmission [9,10]. Potential impact would depend on whether animal health and laboratory signals are rapidly connected with vector, environmental, movement pathway, and occupational exposure information, with vector competence providing one component of this preparedness problem [30].

Probability and consequence are different dimensions of RVF risk. A very low estimated probability of RVFV introduction can coexist with high potential impact if recognition is delayed in immunologically naive European livestock populations. Delayed recognition could permit animal amplification and occupational exposure before control measures are fully activated. The practical burden would then fall on movement restrictions, carcass and abortion material management, vaccination or culling decisions, zoning, and vector control. Preparedness should therefore be proportionate but operational: routine animal health vigilance and diagnostic access are maintained at baseline, while compatible livestock events, laboratory signals, credible movement or product-entry alerts, concentrated receptivity near susceptible livestock, or confirmed multisector evidence trigger escalation.

Operationally, the conditional convergence framework functions as a staged decision sequence for European RVF preparedness. During baseline periods, proportionate investment lies in professional awareness, differential diagnosis of abortion and neonatal mortality, laboratory access, movement–route awareness and seasonal mapping in receptive livestock–vector interfaces [54,55]. Once animal, human, vector or slaughterhouse signals emerge, diagnostic readiness determines whether molecular and serological evidence can distinguish acute infection from previous exposure [51,52]. Targeted investigation is warranted when indirect signals converge in place and time. Examples include compatible livestock disease after a movement concern or increased vector abundance during a suitable season [44,54]. Direct RVFV detection or acute animal or human evidence supported by movement and vector–environment context should move the response to emergency control [56,57]. At that stage, biosecurity, movement control and vaccination decisions should follow the legal and livestock-control framework [68]. One Health exercises are most valuable when they test this escalation sequence before an event [67,69].

**Figure 3:**
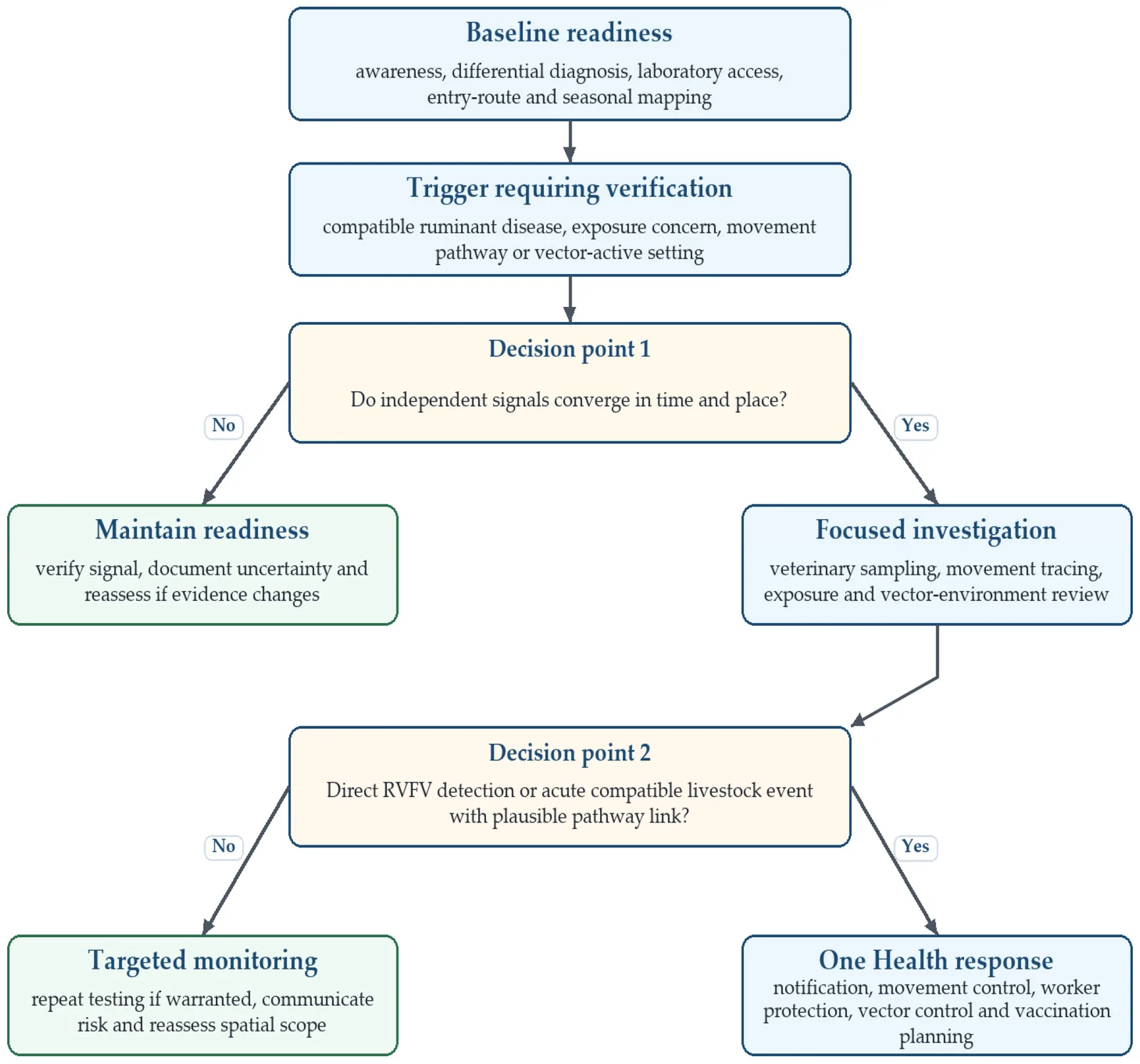
Conditional convergence decision tree for European RVFV preparedness. The pathway shows how isolated signals support verification, how convergent signals justify focused investigation, and how direct RVFV detection or acute pathway-linked livestock events support coordinated One Health response.

Figure 3 operationalizes the conditional convergence framework as a decision pathway linking isolated signals, convergent evidence, focused investigation, and coordinated One Health response.

Together, this sequence turns conditional convergence into a resource allocation rule. Low-cost baseline readiness is maintained broadly, spatially targeted surveillance is concentrated where livestock–vector interfaces raise decision value, focused investigation follows convergent indirect signals, and cost-intensive multisector response is reserved for direct RVFV evidence or acute compatible evidence with high expected intervention value. This balance is consistent with EU assessments of low baseline probability [11], European high-consequence preparedness analyses [9,10], vector competence evidence [30], EFSA entry-route evidence [39] and EFSA control modeling [44]. The practical contribution is to align pathway reduction, sampling thresholds, and coordinated movement control or vaccination decisions within one proportionate preparedness logic [56,57].

## Figures and Tables

**Table 1:** Evidence-to-inference framework used to appraise heterogeneous RVFV evidence in this narrative review.

Evidence Type	Study Design/Source	Geographical Context/Population or Vector Species Examined	Endpoint Assessed	Assumptions	Key Limitations	Certainty for European Preparedness
Direct virological confirmation	Virus isolation, RT-PCR/RT-qPCR, IgM-supported acute investigation	Animals, humans, or vectors in outbreak or surveillance settings	Active/recent infection or viral detection	Sampling captures the relevant viraemic or infectious window	Short detection window; negative results do not exclude earlier exposure or later amplification	High for circulation when epidemiologically linked; moderate when isolated or poorly contextualized
Serological surveys	Cross-sectional or surveillance serology	Livestock, wildlife, camels, or humans	IgG/total antibodies, IgM where reported	Assays are specific, and denominators represent the target population	IgG mainly indicates past exposure; timing, cross-reactivity, sampling frame, and denominator affect interpretation	Moderate for exposure; low for active transmission unless supported by IgM, PCR, or outbreak data
Outbreak investigations	Field investigation, animal health reporting, human case investigation, slaughterhouse or sentinel surveillance	Endemic, epidemic, or interface settings	Clinical impact, animal amplification, occupational exposure, response performance	Detected cases reflect a wider epidemiological event	Underreporting, delayed detection, and setting-specific health-system capacity limit transferability	High for impact and operational lessons; conditional for European prediction
Experimental vector competence	Laboratory infection, dissemination, saliva/transmission, or host-to-vector experiments	Defined mosquito species or populations under controlled conditions	Infection, dissemination, saliva positivity, transmission proxy, or mosquito infection after feeding on viraemic hosts	Laboratory endpoints approximate biological plausibility	Species, colony/field origin, temperature, dose, feeding system, incubation period, and endpoint definitions affect cross-study comparability	Moderate for biological plausibility; low for field transmission without introduction and animal/vector field evidence
Vector persistence and vertical transmission evidence	Laboratory studies and host–vector or between-season models	Specific mosquito species and vertebrate–host systems	Transovarial/vertical transmission, progeny infection, persistence thresholds	Vector-associated maintenance can contribute to interepidemic persistence	Evidence is species-specific and may not generalize to European vector populations or field ecology	Limited to moderate for persistence plausibility; low for European establishment without local confirmation
Ecological suitability and spatial models	Environmental suitability mapping and spatial risk analysis	Europe, Mediterranean, Africa, Middle East, or country-level grids	Receptivity, habitat suitability, seasonal or spatial hazard	Environmental covariates can identify where surveillance may be more efficient	Modeled suitability helps prioritize investigation, but inference on RVFV circulation requires direct virological, animal health, or linked epidemiological evidence; model outputs remain sensitive to occurrence data, covariates, spatial scale, and assumptions	Moderate for prioritizing surveillance areas; low for proving emergence
Dynamic transmission and control models	Host–vector transmission, spread, persistence, or intervention models	Livestock–vector systems and scenario-based European settings	R0, growth rate, spread radius, persistence, intervention sensitivity	Parameterization captures relevant host, vector, and seasonal processes	Outputs vary with vector-to-host ratio, introduction timing, dispersal, surveillance sensitivity, and control assumptions	Moderate for scenario planning; conditional for operational decisions
Institutional risk assessments	EFSA/ECDC/WOAH/WHO/FAO or national assessments and guidance	Regulatory, surveillance, and preparedness settings	Introduction pathway, control feasibility, surveillance option, policy implication	Risk estimates reflect current rules, reporting systems, and available evidence	Not primary field evidence; conclusions may change with trade, climate, vector, surveillance, or policy changes	High for regulatory context; conditional for local preparedness decisions

**Table 3:** Scenario-based preparedness framework for RVFV surveillance, investigation, and response in a European One Health context.

Evidence Type	Study Design/Source	Geographical Context/Population or Vector Species Examined	Endpoint Assessed	Assumptions	Key Limitations	Certainty for European Preparedness
Direct virological confirmation	Virus isolation, RT-PCR/RT-qPCR, IgM-supported acute investigation	Animals, humans, or vectors in outbreak or surveillance settings	Active/recent infection or viral detection	Sampling captures the relevant viraemic or infectious window	Short detection window; negative results do not exclude earlier exposure or later amplification	High for circulation when epidemiologically linked; moderate when isolated or poorly contextualized
Serological surveys	Cross-sectional or surveillance serology	Livestock, wildlife, camels, or humans	IgG/total antibodies, IgM where reported	Assays are specific, and denominators represent the target population	IgG mainly indicates past exposure; timing, cross-reactivity, sampling frame, and denominator affect interpretation	Moderate for exposure; low for active transmission unless supported by IgM, PCR, or outbreak data
Outbreak investigations	Field investigation, animal health reporting, human case investigation, slaughterhouse or sentinel surveillance	Endemic, epidemic, or interface settings	Clinical impact, animal amplification, occupational exposure, response performance	Detected cases reflect a wider epidemiological event	Underreporting, delayed detection, and setting-specific health-system capacity limit transferability	High for impact and operational lessons; conditional for European prediction
Experimental vector competence	Laboratory infection, dissemination, saliva/transmission, or host-to-vector experiments	Defined mosquito species or populations under controlled conditions	Infection, dissemination, saliva positivity, transmission proxy, or mosquito infection after feeding on viraemic hosts	Laboratory endpoints approximate biological plausibility	Species, colony/field origin, temperature, dose, feeding system, incubation period, and endpoint definitions affect cross-study comparability	Moderate for biological plausibility; low for field transmission without introduction and animal/vector field evidence
Vector persistence and vertical transmission evidence	Laboratory studies and host–vector or between-season models	Specific mosquito species and vertebrate-host systems	Transovarial/vertical transmission, progeny infection, persistence thresholds	Vector-associated maintenance can contribute to interepidemic persistence	Evidence is species-specific and may not generalize to European vector populations or field ecology	Limited to moderate for persistence plausibility; low for European establishment without local confirmation
Ecological suitability and spatial models	Environmental suitability mapping and spatial risk analysis	Europe, Mediterranean, Africa, Middle East, or country-level grids	Receptivity, habitat suitability, seasonal or spatial hazard	Environmental covariates can identify where surveillance may be more efficient	Modeled suitability helps prioritize investigation, but inference on RVFV circulation requires direct virological, animal health, or linked epidemiological evidence; model outputs remain sensitive to occurrence data, covariates, spatial scale, and assumptions	Moderate for prioritizing surveillance areas; low for proving emergence
Dynamic transmission and control models	Host–vector transmission, spread, persistence, or intervention models	Livestock–vector systems and scenario-based European settings	R0, growth rate, spread radius, persistence, intervention sensitivity	Parameterization captures relevant host, vector, and seasonal processes	Outputs vary with vector-to-host ratio, introduction timing, dispersal, surveillance sensitivity, and control assumptions	Moderate for scenario planning; conditional for operational decisions
Institutional risk assessments	EFSA/ECDC/WOAH/WHO/FAO or national assessments and guidance	Regulatory, surveillance, and preparedness settings	Introduction pathway, control feasibility, surveillance option, policy implication	Risk estimates reflect current rules, reporting systems, and available evidence	Not primary field evidence; conclusions may change with trade, climate, vector, surveillance, or policy changes	High for regulatory context; conditional for local preparedness decisions

## Data Availability

No new data were created or analyzed in this study. Data sharing is not applicable to this article.
